# The Role of Tumor Associated Macrophages in Hepatocellular Carcinoma

**DOI:** 10.7150/jca.51346

**Published:** 2021-01-01

**Authors:** Yu Huang, Wenhao Ge, Jiarong Zhou, Bingqiang Gao, Xiaohui Qian, Weilin Wang

**Affiliations:** 1 Department of Hepatobiliary and Pancreatic Surgery, The Second Affiliated Hospital, Zhejiang University School of Medicine, Hangzhou, Zhejiang 310009.; 2 Key Laboratory of Precision Diagnosis and Treatment for Hepatobiliary and Pancreatic Tumor of Zhejiang Province, Hangzhou, Zhejiang 310009.; 3 Research Center of Diagnosis and Treatment Technology for Hepatocellular Carcinoma of Zhejiang Province, Hangzhou, Zhejiang 310009.; 4 Clinical Medicine Innovation Center of Precision Diagnosis and Treatment for Hepatobiliary and Pancreatic Disease of Zhejiang University, Hangzhou, Zhejiang 310009.; 5 Clinical Research Center of Hepatobiliary and Pancreatic Diseases of Zhejiang Province, Hangzhou, Zhejiang 310009.

**Keywords:** Hepatocellular carcinoma, Tumor associated macrophage, Tumor microenvironment, Immunotherapy

## Abstract

Hepatocellular carcinoma (HCC) is one of the most common cancers worldwide and represents a classic paradigm of inflammation-related cancer. Various inflammation-related risk factors jointly contribute to the development of chronic inflammation in the liver. Chronic inflammation, in turn, leads to continuous cycles of destruction-regeneration in the liver, contributing to HCC development and progression. Tumor associated macrophages are abundant in the tumor microenvironment of HCC, promoting chronic inflammation and HCC progression. Hence, better understanding of the mechanism by which tumor associated macrophages contribute to the pathogenesis of HCC would allow for the development of novel macrophage-targeting immunotherapies. This review summarizes the current knowledge regarding the mechanisms by which macrophages promote HCC development and progression, as well as information from ongoing therapies and clinical trials assessing the efficacy of macrophage-modulating therapies in HCC patients.

## 1. Introduction

Hepatocellular carcinoma (HCC) is one of the most common cancers worldwide 1. Despite advances in surgical resection, adjuvant therapy, and liver transplantation during the past decades, the survival rate of HCC patients remains unsatisfactory, with a 5-year survival rate of approximately 20%. The poor prognosis of HCC is primarily due to its late diagnosis, as well as its high risk of potential recurrence and metastasis 2,3. Other non-surgical interventions, including transarterial chemoembolization (TACE) and radiofrequency ablation (RFA), can provide disease control only for advanced-stage patients 4. Systemic administration of sorafenib exerts a weak therapeutic effect in patients with metastatic HCC 5. Therefore, the development of novel therapeutics with mechanisms different from those of the currently available treatments is crucial to improve the prognosis of HCC patients.

Emerging immunotherapies, including immune checkpoint inhibitors targeting programmed death-1 (PD-1) and cytotoxic T lymphocyte-associated antigen 4 (CTLA4), have revolutionized the therapeutic landscape for various solid tumors 6-9. HCC is a typical example of inflammation-related cancer. Primary risk factors for HCC include exposure to aflatoxins, alcohol intoxication, non-alcoholic fatty liver disease caused by obesity and metabolic syndrome, as well as viral infections such as hepatitis B and C viruses. These factors jointly contribute to hepatic inflammation, fibrosis, and cirrhosis, which are observed in 80% of HCC patients 10-12. In the inflammatory tumor microenvironment (TME) of HCC, various non-malignant cells and extracellular matrix components play pivotal roles in cancer development and progression, as well as in resistance to traditional antitumor therapies 13,14.

TME is characterized by infiltration of various non-malignant cells, including stromal cells, fibroblasts, macrophages, endothelial cells, immune cells, and circulating platelets. Cytokines secreted by these cells and the extracellular matrix are also important components of the TME 15-17. Within the TME, non-malignant cells play crucial roles in promoting cancer cell proliferation, invasion, and metastasis. Among them, immune cells coexist and interact with each other initiating complex pathways and ultimately resulting in tumor progression and drug resistance. Thus, the combination of immunological interventions with conventional therapies might be more effective than monotherapies 18,19. Macrophages are major components of the TME; several tumor-promoting roles have been attributed to these tumor associated macrophages (TAMs) 20-23. TAMs have been implicated in immune suppression, cancer invasion and metastasis, angiogenesis, maintenance of cancer cell stemness, and drug resistance. Furthermore, high levels of TAMs have been associated with poor prognosis in patients with HCC 24-26. Therefore, a better understanding of the mechanisms underlying the function of TAMs is necessary for the development of novel TAM-targeting immunological interventions, which may provide promising therapeutic approaches for HCC patients 27-29.

This review summarizes the current knowledge regarding the role of macrophages in the pathogenesis of HCC and provides information regarding the currently available immunotherapies as well as ongoing clinical trials assessing the efficacy of macrophage-modulating therapies in HCC patients.

### 1.1. Liver macrophages in homostasis

As one of the main types of innate immune cells, macrophages serve as the first line of defense against pathogenic insults to the body. Macrophages can be found in all tissues and exhibit exceptionally high plasticity and functional diversity 30-32. Macrophages are involved in phagocytosis, antigen processing and presentation, and orchestration of the immune system by the release of multiple cytokines, regulating inflammation initiation, progression, and resolution 33,34.

**Figure 1:**
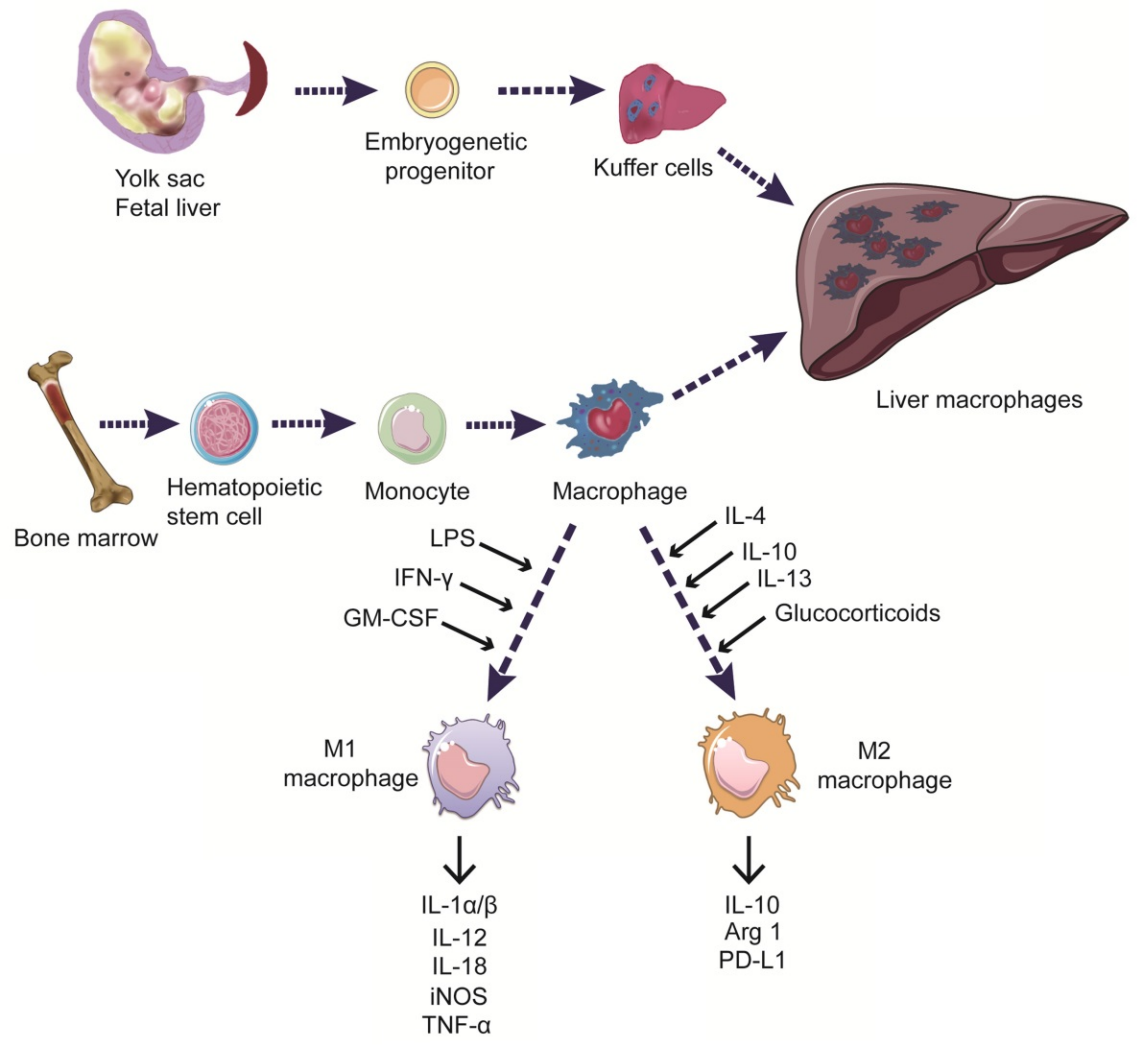
** Origin and classification of liver macrophages.** Liver macrophages, consisting of Kuffer cells and monocyte-derived macrophages, play essential roles in sustaining hepatic and systematic homeostasis. Myeloid cells including blood monocytes, scan the liver vasculature and eventually derive macrophages infiltrating into the liver. Kuffer cells are resident phagocytes originating from yolk sac-derived precursors during embryogenesis. Macrophages can be classified into two distinct functional phenotypes according to their responses to different environmental stimuli *in vitro*. M1 macrophages are induced by LPS, IFN-γ, and GM-CSF. They secrete the pro-inflammatory cytokines IL-1α/β, IL-12, IL-18, iNOS, and TNF-α, as well as promoting the function of effector T cells. M2 macrophages are induced by IL-4, IL-10, IL-13, and glucocorticoids, and exert anti-inflammatory and immune-regulatory effects by expressing IL-10, Arg1, and PD-L1.

The liver harbours the main part body macrophages and is supervised by myeloid cells including blood monocytes, which scan the liver vasculature and eventually infiltrate into the liver. Under homeostasis conditions, monocyte-derived cells can develop into liver dendritic cells (DCs) or monocyte-derived macrophages (MoMFs), the latter do not contribute to the pool of resident macrophages, termed Kuffer cells. Kuffer cells originate from yolk sac-derived precursors during embryogenesis, forming a self-renewing pool of resident macrophages in the liver and playing essential roles in sustaining hepatic and systematic homeostasis 35. When activated by danger signals, Kuffer cells can promote chronic liver inflammation by inducing the recruitment of immune cells to the liver, including monocytes, which subsequently derived DCs and MoMFs 36. To date, no specific markers were proved to distinguish human Kuffer cells from monocyte-derived cells (Figure 1).

Additionally, according to their inflammatory states in response to different environmental stimuli, macrophages can be classified into two main subtypes: the classically activated macrophages (M1 macrophages) and alternatively activated macrophages (M2 macrophages). These two distinct functional phenotypes have contradicting roles in regulating inflammation progression *in vitro* 37,38. M1 macrophages are primarily induced by microbial components, such as lipopolysaccharides (LPS), or by pro-inflammatory cytokines, including interferon-γ (IFN-γ), tumor necrosis factor (TNF), and toll-like receptor (TLR) ligands. M1 macrophages exert pro-inflammatory functions by releasing nitric oxide (NO), reactive oxygen species (ROS), and the pro-inflammatory cytokines interleukin (IL)-1, IL-6, IL-12, TNF-α, CXCL5, and CXCL8-10, involving in antigen processing and presentation, promoting the function of effector T cells 39,40. Polarization to the M2 phenotype is induced by IL-4, IL-10, and IL-13, as well as by glucocorticoids. M2 macrophages exert immunosuppressive functions and promote tissue repair by secreting IL-10 and other immunosuppressive cytokines 41,42 (Figure 1). However, there are almost none of the purely M1 or M2 macrophages in scenarios like tumor models or autoimmune disease *in vivo*. Thus, more research for a better understanding of the relationship between mechanisms and the subphenotypes of macrophages is urgently needed in developing novel therapies.

## 2. Liver macrophages in pathogenesis of HCC

Increased infiltration of macrophages is a common characteristic of various solid tumors. Concurrently, HCC represents a classic paradigm of inflammation-related cancer. Various inflammation-related risk factors jointly contribute to the development of chronic inflammation in the liver. Chronic inflammation, in turn, leads to continuous cycles of destruction-regeneration in the liver, contributing to fibrosis and cirrhosis, and eventually development and progression of HCC. Liver macrophages are regulators of this process 43,44. An immunogenic analysis using patient data from The Cancer Genome Atlas indicated that TAMs are abundant in HCC, which are mostly polarized towards the M2 phenotype. CD68 is commonly used as an indicator of liver TAMs, and the expression levels of CD86 (M1), CD163 (M2), and CD206 (M2) are widely accepted to differentiate between M1 and M2 macrophages *in vitro* 45. Low levels of CD86^+^ M1 macrophages and high levels of CD206^+^ M2 macrophages have been associated with an aggressive phenotype in HCC, suggesting that conjoint analysis of CD86 and CD206 expression may provide a prognostic tool for HCC 46. Numerous chemokines (CCL2, CCL5, CCL15, CCL20), cytokines (such as CSF-1) and other products of the complement cascade were demonstrated to participate in the mechanism of monocyte-derived macrophages recruitment and migration 23,47-49. Additionally, several researches recently provide evidence in the transition from Kuffer cells to TAM pool by triggering Her2/Neu pathway 50,51. Other factors, including mitochondrial DNA (mtDNA), osteopontin (OPN), micro RNAs (miRNAs), circular RNAs (circRNAs) and HCC cell-derived exosomes were also reported to play important roles in TAM recruitment 52-55. TAMs induce the expression of ST18 in epithelial cells, promoting mutual epithelial cell-macrophage dependency in HCC 56. TAMs also release various immunosuppressive chemokines and cytokines, including IL-10, transforming growth factor beta (TGF-β), which exert immunoregulatory roles. Furthermore, TAMs have been shown to recruit regulatory T cells (Tregs) to the tumor; the recruitment of Tregs impairs the activation and function of effector T cells 57.

Recent studies have indicated that insulin-like growth factor (IGF)-1 and IGF-2 remodel macrophages during their maturation 58. Tricarboxylic acid cycle metabolism plays a key role in the epigenetic remodeling of macrophages 59. In mouse models of glioblastoma, IGF-1 enhanced PI3K-mediated tumor cell proliferation in a macrophage-dependent manner 60. Nevertheless, the molecular mechanisms underlying the IGF-mediated reprogramming of macrophages in human HCC require further investigation.

**Figure 2:**
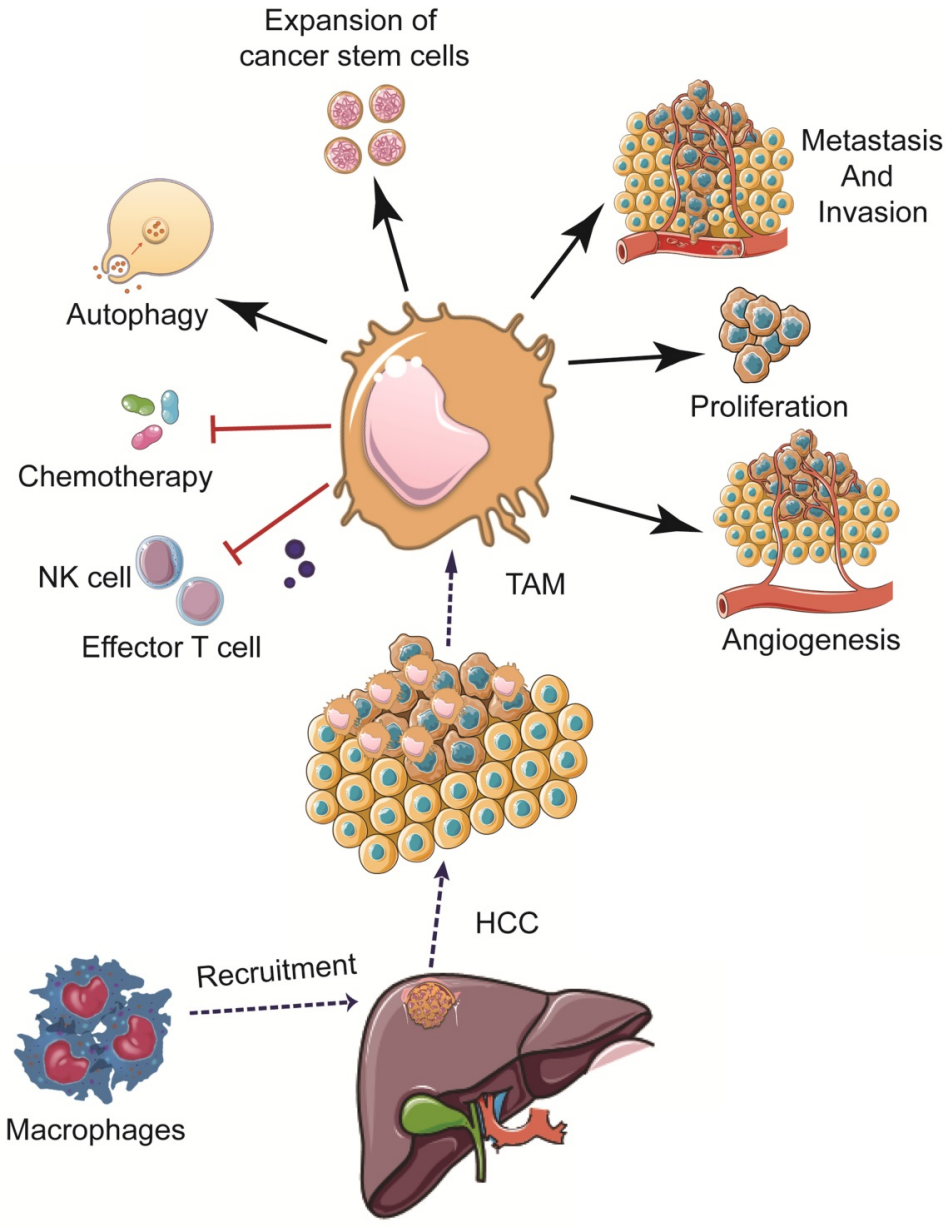
** Effects of TAMs in the pathogenesis of HCC.** TAM can promote HCC development and progression via multiple mechanisms, including promoting cancer cell proliferation, stemness, invasion, and metastasis, modulating angiogenesis, autophagy, drug resistance, as well as weakening the functions of NK cells and effector T cells, etc.

Increased levels of TAMs have been shown to promote angiogenesis, cancer cell proliferation, invasion, and metastasis; high levels of TAMs have also been associated with a poor prognosis in HCC patients 61,62. The mechanisms of TAMs in the pathogenesis of HCC are summarized hereinbelow (Figure 2).

### 2.1. TAMs promote cancer cell proliferation, invasion, and metastasis in HCC

M2 macrophages have been shown to play an essential role in promoting cancer cell migration in HCC via the TLR4/STAT3 signaling pathway 63. Aberrant activation of the NTS/IL-8 pathway was reported to play a pro-tumorigenic role in the inflammatory microenvironment of HCC, by augmenting M2 macrophage-mediated EMT of cancer cells 64. CXCL8 produced by activated macrophages promotes HCC progression and metastasis 65. TIM-3 augments the TGF-β mediated polarization of macrophages toward an M2 phenotype, contributing to the poor prognosis of HCC 66. IL-6 derived by TAMs has been suggested to promote cancer cell invasion, and metastasis in HCC 67. SPON2 has been reported to promote the infiltration of M1 macrophages in HCC and suppress metastasis via the integrin/RhoGTPase/Hippo pathway 68. MicroRNAs, as a class of small non-coding RNAs, regulating gene expression at the post-transcriptional level, were reported to mediate tumor-promoting effects of TAMs recently. Downregulation of miR-28-5p expression in HCC samples has been inversely associated with the levels of TAM infiltration and IL-34 expression; IL-34 further promotes TAM infiltration, resulting in a miR-28-5p-IL-34 feedback loop, which plays an important role in HCC metastasis 69. MiR-98 has been shown to suppress cancer cell invasion in HCC by promoting macrophage polarization from the M2 to M1 phenotype 70. MiR-146a-5p, enriched in HCC cell-derived exosomes, was demonstrated to promote infiltration of M2 TAMs, which results in T cell exhaustion and HCC progression 54. Coincidentally, long non-coding RNAs also play an important role in the tumor-promoting effects of macrophages in HCC. Long non-coding RNA COX-2 suppresses immune evasion and metastasis in HCC by inhibiting macrophage polarization into an M2 phenotype 71. Recently, using single-cell RNA sequencing, RIPK1 was demonstrated to induce CCR2^+^ macrophages infiltration, as well as promoting liver fibrosis and hepatocarcinogenesis 72. Wu *et al*. recently manifested that CD11b/CD18, as well as integrin, which derived from M2 macrophage exosomes, had the potency to boost the migratory potential of HCC cells 73. Additionally, HCC cell-derived Wnt ligands stimulate M2 polarization of TAMs, which reversely result in tumor growth, metastasis and immunosuppression in HCC 74. Heat shock transcription factor 1 (HSF1) was demonstrated as a vital mediator in metabolic alteration of HCC cells in cross-talking with TAMs 75.

Hypoxia has been demonstrated to mediate the effects of macrophages, playing an important role in HCC, among other solid tumors. Hypoxia-induced EMT increased the expression of CCL20 in hepatoma cells, leading to indoleamine 2,3-dioxygenase (IDO) upregulation in monocytes-derived macrophages. Macrophages that are derived from IDO+ monocytes counteract effector T cells and promote tolerance to tumor antigens 76. Zhang *et al*. found that the necrotic debris from cancer cells in the hypoxic and inflammatory HCC microenvironment enhanced the release of IL-1β by M2 macrophages. IL-1β, in turn, unregulated the expression of HIF-1α in HCC cells in a cyclooxygenase-2-dependent manner, constituting a positive feedback loop promoting EMT in HCC cells 77. Wu *et al*. confirmed that TREM-1^+^ TAMs promote the recruitment of CCR6^+^Foxp3^+^ Tregs via the ERK/NF-κB pathway, conferring resistance to programmed cell death ligand 1 (PD-L1) treatment in HCC 78.

### 2.2. TAMs promote angiogenesis in HCC

TAMs have been demonstrated to produce numerous angiogenic factors, including vascular endothelial growth factor, platelet-derived growth factor, and several matrix metalloproteinases 79. The CCR2^+^ TAM subset was reported to be highly enriched in highly vascularized HCC tumors and was suggested to drive angiogenesis and tumor vascularization in fibrotic livers 80. A recent study showed that HCC patients with high serum levels of IL-23 derived from macrophages had tumors with enhanced vascularization 81. CXCR4, expressing on endothelial cells, which is upregulated by inflammatory cytokines derived from TAMs via ERK pathway activation, was identified as a novel vascular marker in HCC tissues 82. The combination of sorafenib and zoledronic acid (ZA) has been shown to exert synergistic antitumor effects mediated by downregulation of CXCR4 expression 83.

### 2.3. TAMs promote cancer cell stemness in HCC

Previous studies have suggested that the high heterogeneity and malignancy of HCC are partly attributed to cancer stem cells (CSCs), which promote tumor recurrence, metastasis, and development of resistance to therapies 84. Numerous cell surface proteins, including EPCAM, CD133, CD124, CD44, and CD90, have been identified as CSC markers 85,86. TAMs have been suggested to promote CSC-like properties via various signaling pathways. Importantly, TAMs facilitated the expansion of stem cells via the IL-6/STAT3 pathway in HCC patients 87. Fan *et al*. confirmed that TAMs promote CSC properties via TGF-β signaling pathway 88. TNF-α promotes EMT and cell stemness in HCC by activating the Wnt/β-catenin signaling pathway, which can be partially reversed by the Wnt/β-catenin inhibitor ICG-001 89. In addition, exosomes from TAMs promote cancer cell proliferation and stem cell properties in HCC. For instance, a low level of miR-125a/b in exosomes from TAMs was shown to inhibit CSCs by targeting CD90 in HCC 90.

### 2.4. TAMs promote autophagy in HCC

Mounting evidence suggests the importance of autophagy in the regulation of the function of TAMs and antitumor immunity 91. A recent study showed that autophagy-deficient Kupffer cells promoted liver fibrosis, inflammation, and hepatocarcinogenesis via the mitochondrial reactive oxygen species/NF-κB/IL-1α/β signaling axis 92. Chang *et al*. demonstrated that TLR2 related ligands triggered NFKB RELA cytoplasmic ubiquitination and led to its degradation by SQSTM1/p62-mediated autophagy, promoting M2 polarization in macrophages and immunosuppression in HCC 93. TLR2 deficiency also resulted in a decrease in macrophage infiltration and inhibition of apoptosis signal-regulating kinase 1 and p38 mitogen-activated protein kinase/NF-κB signaling. Subsequently, TLR2 deficiency led to decreased expression levels of IFN-γ, TNF-α, and IL-1α/β, as well as increased cell proliferation and suppressed autophagy and apoptosis in mouse liver cells 94. Tan *et al*. revealed that the natural compound baicalin inhibited HCC development and progression by TAM repolarization towards the M1 phenotype via autophagy-associated activation of RelB/p52 95. Fu *et al*. suggested that TAMs induce autophagy in HCC cells, which might contribute to oxaliplatin resistance 24. These findings reveal new roles of TAMs and autophagy in HCC, which could provide new opportunities for the development of more efficient therapies.

### 2.5. TAMs modulate therapeutic resistance in HCC

The orally administrated multikinase inhibitor sorafenib shows limited efficacy in HCC patients due to the development of intolerance and resistance 96. TAM has been demonstrated to induce immunosuppression and weaken the efficacy of sorafenib in HCC 97. Zhou *et al*. demonstrated that tumor-associated neutrophils enhance the recruitment of Tregs and macrophages to the TME in HCC patients, promoting tumor progression and resistance to sorafenib 98. Oxaliplatin-based chemotherapies are widely used in patients with advanced HCC. It has been reported that TAMs are important drivers of resistance to oxaliplatin by trigging autophagy and apoptosis evasion in HCC cells 24. The density of TAMs in HCC samples has been associated with the efficiency of transarterial chemoembolization in HCC 24. Moreover, M2 macrophages have been reported to promote the development of resistance to sorafenib in HCC by secreting hepatocyte growth factor (HGF) 25. A recent study showed that sorafenib induces pyroptosis in macrophages and facilitates NK cell-mediated cytotoxicity in HCC, highlighting the importance of TAMs as a therapeutic target in HCC 99.

## 3. Macrophage-targeting therapies in HCC

**Figure 3:**
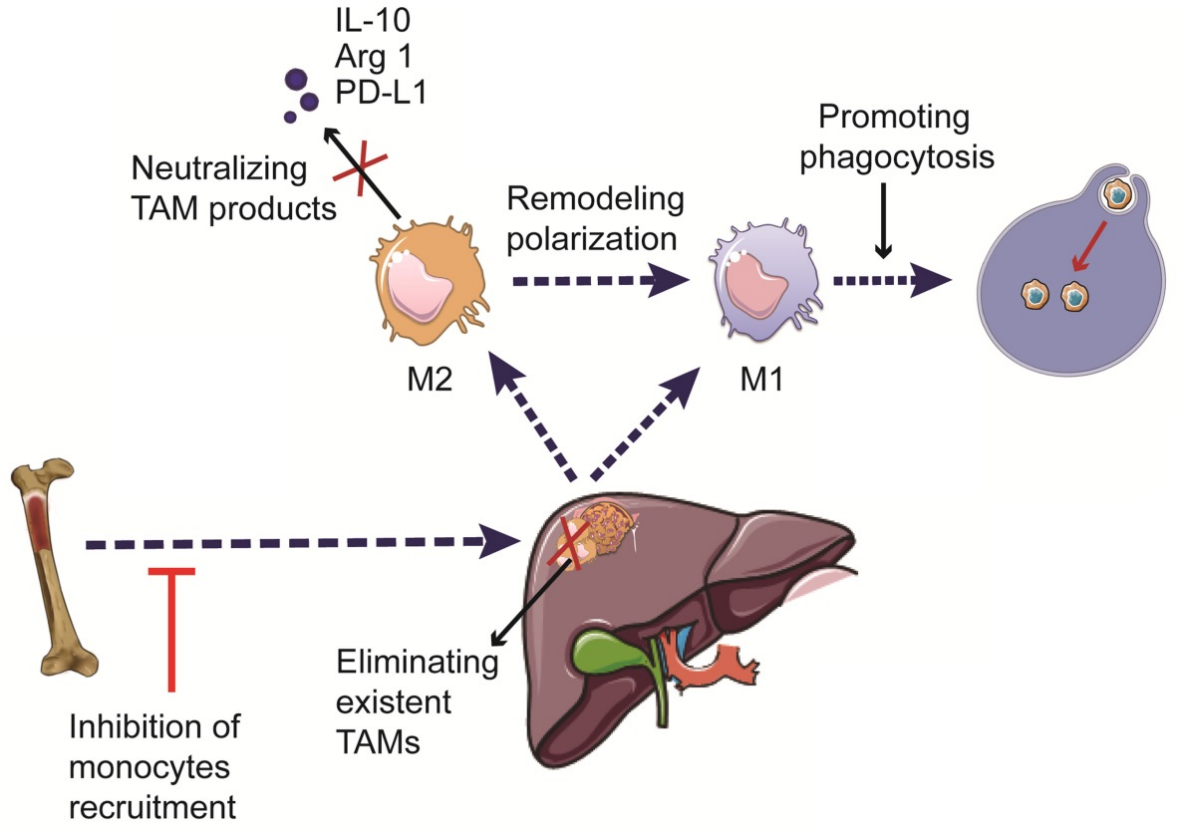
** Therapeutic strategies targeting TAMs in HCC.** The development of TAM-targeting immunotherapeutic strategies includes induction of phagocytosis, inhibition of monocyte recruitment, elimination of pre-existing TAMs, reprogramming of macrophage polarization, and neutralization of the pro-tumorigenic factors secreted by TAMs.

Increasing evidence suggests the critical roles of TAMs in HCC development and progression. Hence, immunotherapies targeting TAMs have emerged as a promising approach to treat patients with HCC. The current therapeutic strategies targeting TAMs include phagocytosis-promoting therapies, inhibition of monocyte recruitment, elimination of pre-existing TAMs in the tumor tissue, remodeling TAM polarization, and neutralizing pro-tumorigenic factors secreted by TAMs 100,101 (Figure 3). The currently available immunotherapies, as well as ongoing clinical trials involving the use of TAM-targeting immunotherapies in HCC, are summarized in Tables 1 and 2.

### 3.1. Phagocytosis-promoting therapies

CD47, mostly known as a receptor for thrombospondin in human myeloid and endothelial cells, has recently been demonstrated to protect host cells from macrophage-mediated destruction by binding to SIRP1α (SHPS-1) expressed on the surface of macrophages 102. Neutralizing antibodies against CD47 can enhance macrophage-mediated phagocytosis and activate effector T cells 103,104. IL-6 secretion by TAMs has been reported to upregulate CD47 expression in HCC cells via the STAT3 signaling pathway. CD47 upregulation has been associated with poor overall survival and recurrence-free survival in HCC patients. Blockage of CD47 enhanced TAM-mediated phagocytosis in the presence of chemotherapeutic agents 105. Yang *et al*. demonstrated that the HDAC6/let-7i-5p/TSP1 axis reduced the neoplastic and antiphagocytic properties of HCC cells by targeting CD47, providing a promising therapeutic target for the treatment of HCC 106. Additionally, the anti-CD47 monoclonal antibody (B6H12) suppressed tumor growth and augmented the efficacy of chemotherapy in HCC 107,108.

### 3.2. Therapies inhibiting the recruitment of macrophages

The inhibition of monocyte recruitment in HCC tissues has recently emerged as a promising approach to decrease the levels of TAMs. Aberrant expression of miR-26a has been shown to suppress HCC growth and macrophage infiltration by targeting macrophage colony-stimulating factor (M-CSF) in a PI3K/AKT pathway-dependent mechanism 109. CCL2 expression has been reported to be elevated in HCC tissues and has been suggested as a novel prognostic factor for HCC. Kuffer cells, as the primary source of CCL2, are essential for the recruitment and education of monocyte-derived macrophages 36. The CCL2/CCR2 signaling has recently emerged as a target to suppress the recruitment of monocytes in tumors. Blockade of CCL2/CCR2 signaling pathway suppressed monocyte recruitment and the polarization of infiltrated macrophages towards the M2 phenotype, suppressing tumor growth in a T cell-dependent manner, in a mouse liver cancer model 110. The CCR2 antagonist 747 has been shown to exert potent antitumor effects and enhance the efficacy of sorafenib by combating TAM-mediated immunosuppression and increasing the number of CD8+ T cells in an HCC mouse model 97. Glypican-3 has been reported to be overexpressed in HCC and has been implicated in the recruitment of macrophages by binding to CCL3 and CCL5 111,112. Antibodies targeting glypican-3 have demonstrated promising efficacy in several clinical trials by inhibiting the recruitment of M2 macrophages in the TME 113. For example, the humanized antibody GC33 was well tolerated in advanced HCC patients in a clinical trial conducted in Japan 114.

### 3.3. Therapies eliminating pre-existing TAMs

The combination of TAM-targeting therapies with traditional therapies in HCC has been investigated 21. For instance, clodrolip or ZA treatment augmented the antitumor effects of sorafenib, by suppressing tumor growth, angiogenesis, and lung metastasis in HCC xenograft models 83. The use of ZA may remodel the intratumoral pool of macrophages by trigging apoptosis in specific TAM populations 115,116. In addition, ZA treatment has been shown to enhance the effects of transarterial chemoembolization by suppressing the infiltration of TAMs in HCC 117.

### 3.4. Reprogramming TAM polarization

Numerous tumor-derived factors are involved in TAM phenotype shift, including the aforementioned TGF-β, miR-98, and long non-coding RNA COX-2 66,70,71. Recently, it is reported that HCC-derived exosomes could remodel macrophages by activating NF-κB signaling and result in M2 polarized TAMs 54. Receptor-interacting protein kinase 3 (RIPK3) is downregulated in HCC-associated macrophages, and RIPK3 deficiency induced fatty acid oxidation (FAO), which induced M2 polarized TAMs. Hence, RIPK3 upregulation or FAO blockade reversed the immunosuppressive activity of TAMs and dampened HCC tumorigenesis 118. As mentioned previously, TAMs with the M1 phenotype promote tumor cell elimination and degradation. Hence, re-educating TAMs to switch from the M2 to M1 phenotype has been suggested as a therapeutic approach for HCC. Baicalin administration suppressed tumor growth in an orthotopic HCC mouse model by inducing TAM reprogramming towards the M1 phenotype and subsequent secretion of pro-inflammatory cytokines 95. Furthermore, 8-bromo-7-methoxychrysin (BrMC) was shown to attenuate the effects of M2 macrophages by influencing the profile of secreted cytokines and reversing M2 polarization of TAMs 119. The use of the competitive CSF-1R inhibitor PLX3397 suppressed tumor growth in an HCC mouse model by shifting the polarization of TAMs towards the M1 phenotype 120.

## Conclusions

Macrophages play essential roles in orchestrating immune responses; nevertheless, dysregulation of their function has been implicated in HCC, among other solid malignancies. Macrophages are abundant in the TME in HCC, exhibiting double-edged roles in controlling tumorigenesis by modulating immune responses. Importantly, TAMs have been shown to enhance HCC development and progression by promoting immune suppression, cancer cell proliferation, invasion, metastasis, and maintenance of cancer cell stemness. Consequently, a better understanding of the mechanisms by which TAMs regulate HCC malignancy would allow for the development of novel and more effective TAM-targeting HCC therapies. Adjuvant treatment with agents targeting TAMs following conventional hepatectomy or liver transplantation may improve the clinical benefit of the therapies currently used for HCC patients.

## Figures and Tables

**Table 1:** Preclinical agents targeting TAMs for HCC treatment

Author	Agent	Target	Mechanism of action	Result
Lu *et al*.^107^	Anti-CD47mAbs	CD47	Promote phagocytosis of macrophages	Suppress tumor growth and enhance the effect of chemotherapy treatment
Xiao *et al*.^108^	B6H12	CD47	Promote phagocytosis of macrophages	Suppress tumor growth and enhance the effect of chemotherapy treatment
Li *et al*.^110^	RDC018	CCR2	Inhibiting monocytes recruitment	Inhibit HCC growth and metastasis, reduce recurrence
Yao *et al*.^97^	747	CCR2	Inhibiting monocytes recruitment	Anti-cancer properties and enhance the efficacy of sorafenib
Zhu *et al*.^113^	GC33	Glypican-3	Eliminating existent TAMs	Japanese Phase II study for advanced HCC
Ikeda *et al*.^114^	GC33	Glypican-3	Eliminating existent TAMs	Clinical trail for advanced HCC patients
Zhang *et al*.^83^	Clodrolip or Zoledronic acid	-	Eliminating existent TAMs	Enhance the efficacy of sorafenib in mouse models
Zhou *et al*.^117^	Zoledronic acid	-	Eliminating existent TAMs	Enhance the efficacy of TACE in HCC mouse models
Tan *et al*.^95^	Baicalin	-	Re-educating TAMs	Suppress tumor growth
Sun *et al*.^119^	8-Bromo-7-methoxychrysin	CD163	Re-educating TAMs	Impede interaction between HCC stem cells and TAMs
Ao *et al*.^120^	PLX3397	CSF1R	Re-educating TAMs	Suppress tumor growth
Wan *et al*.^87^	Tocilizumab	IL-6 receptor	Neutralizing productsof TAMs	Inhibit TAMs mediated stimulation of HCC stem cells

**Table 2:** Clinical trials targeting TAMs for HCC treatment

Target	Agent	Mechanism	Phase	Clinical trial number
Glypican-3	GC33	Glypican-3 antagonist (eliminating existent macrophages)	Phase 2	NCT01507168
CCR2/5	Nivolumab +BMS-813160 /+BMS-986253	CCR2/5 antagonist (inhibits monocyte/macrophage infiltration)	Phase 2	NCT04123379
CSF1R	Chiauranib	Multi-target inhibitor that suppresses angiogenesis-related kinases and CSF1R; decreases the macrophage differentiation.	Phase 1	NCT03245190

## Abbreviations

HCC: hepatocellular carcinoma
TACE: transarterial chemoembolization
RFA: radiofrequency ablation
PD-1: programmed cell death protein 1
CTLA-4: cytotoxic T lymphocyte associated antigen 4
TME: tumor microenvironment
TAM: tumor associated macrophage
DC: dendritic cell
MoMFs: monocyte-derived macrophages
LPS: lipopolysaccharides
IFN-γ: interferon-γ
TNF: tumor necrosis factor
TLR: toll like receptor
NO: nitric oxide
ROS: reactive oxygen species
IL-1: interleukin 1
CXCL5: C-X-C motif chemokine ligand 5
CCL5: C-C motif chemokine ligand 5
CSF: colony stimulating factor
mtDNA: mitochondrial DNA
miRNAs: micro RNAs
circRNAs: cicular RNAs
TGF-β: transforming growth factor beta
Tregs: regulatory T cells
IGF1: insuline-like growth factor
EMT: epithelial-mesenchymal transition
IDO: indoleamine-2,3-dioxygenase
HIF-1α: hypoxia inducible factor 1α
PD-L1: programmed cell death ligand 1
CXCR4: C-X-C motif chemokine receptor 4
ZA: zoledronic acid
CSC: cancer stem cell
HGF: hepatocyte growth factor
BrMC: 8-bromo-7-methoxychrysin
iNOS: inducible nitric oxide synthase
Arg 1: arginine 1
